# 106 Nursing Documentation Variability Among Burn Centers Using the Burn Navigator

**DOI:** 10.1093/jbcr/irac012.109

**Published:** 2022-03-23

**Authors:** Elsa Coates, Julie A Rizzo, Jose Salinas, Maria Serio-Melvin

**Affiliations:** US Army Institute of Surgical Research, JBSA Fort Sam Houston, Texas; US Army Institute of Surgical Research, Fort Sam Houston, Texas; US Army Institute of Surgical Research, San Antonio, Texas; US Army Institute of Surgical Research, Cibolo, Texas

## Abstract

**Introduction:**

Managing burn fluid resuscitation for large burns is challenging and relies heavily on accurate nursing documentation. The Burn Navigator (BN) is a clinical decision-support system designed to guide clinicians in burn fluid resuscitation. However, data entered into the BN do not auto-populate into the electronic medical record (EMR), thus requiring nurses to document in two systems. We sought to compare differences in nursing documentation of data entries between the EMR and the BN on burn patients with ≥ 20% total body surface area (TBSA) undergoing intravenous (IV) fluid resuscitation.

**Methods:**

Institutional Review Board approval was obtained for a multi-center observational study of burn patients undergoing fluid resuscitation using the BN. Data were collected and analyzed between the EMR and BN entries entered into the REDCap database from 5 American Burn Association (ABA)-verified burn centers. The following variables were analyzed: time of burn injury, weight, TBSA burned, urine output (UOP), and hourly IV crystalloid fluid volume.

**Results:**

Analysis included 296 subjects (of 300 enrolled). Results show no significant difference between burn centers for mean weight (BN 87.02 ± 22.9 kg vs. EMR 87.1 ± 23.3 kg), TBSA (BN 40.71 ± 19.24% vs. EMR 40.97 ± 19.29%), or time of burn injury (< 1 hour). The time of injury recorded in the BN versus EMR was later in 44.6% (n=132) of patients and earlier in 46.4%, (n=138) and the same in 8.8% (n=26) of records. Additionally, in 293 records, there was no significant difference between centers in patient UOP (BN 0.91 ± 0.52 ml/kg/hr vs. EMR 0.91 ± 0.63 ml/kg/hr). One site had a significant difference in hourly fluid rates (Figure) due to a lack of inclusion of pre-hospital fluids.

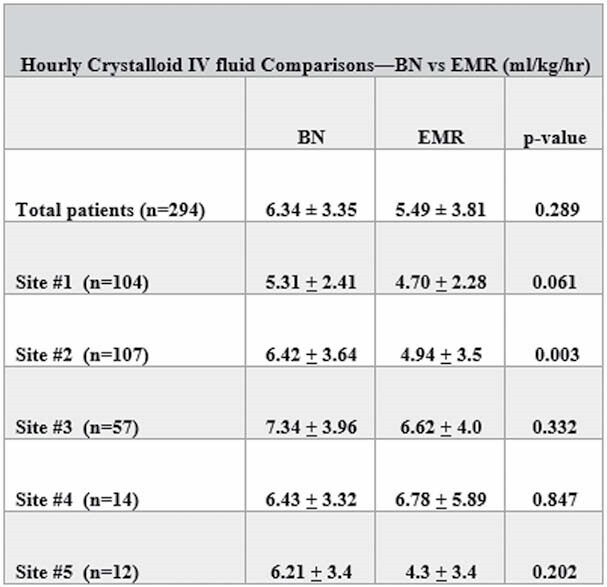

**Conclusions:**

When comparing the data between the EMR to BN, it was observed that pre-hospital fluids tended not to be documented in the EMR, causing a statistically significant difference in total fluids administered in one burn center. Overall, the nursing documentation variability was minimal across all sites even though the nurses had to document the data in two different systems, while simultaneously caring for critically ill patients with large burn injuries. Close monitoring of the nursing documentation during burn fluid resuscitation should always be a priority.

